# Dental caries and related risk factors in Saudi cerebral palsy children

**DOI:** 10.17712/nsj.2017.4.20170191

**Published:** 2017-10

**Authors:** Amjad H. Wyne, Nouf S. Al-Hammad, Christian H. Splieth

**Affiliations:** *From the Department of Pediatric Dentistry and Orthodontics (Wyne, Al-Hammad), College of Dentistry, King Saud University, Riyadh, Kingdom of Saudi Arabia and from the Department of Preventive and Pediatric Dentistry (Splieth), University of Greifswald, Rotgerberstr, Greifswald, Germany*

## Abstract

**Objective:**

To determine caries experience and related risk factors in cerebral palsy (CP) children.

**Methods:**

Random sample of CP children was examined for dental caries and oral hygiene. Questionnaire was utilized for information about caries risk factors. This cross-sectional study was conducted in Riyadh from December 2014 to May 2015.

**Results:**

Fifty-two CP children were examined with mean age of 6.3±2.7 years. Only one (1.9%) child out of the 52 had no clinical caries. Combined (dmft plus DMFT) mean caries score among study sample was 9.98±3.99. Older children had significantly higher mean caries scores (11.5±3.34) than younger children (8.86±4.1, p=0.017). The CP children with good oral hygiene had lowest mean caries score (5.8±7.32) as compared to those with fair (9.72±3.3) and poor (11.55±3.05) oral hygiene (p=0.012). Those children whose first dental visit was for routine check-up had significantly (p=0.02) lower mean caries scores (7.33±4.65) than those who made their first visit due to dental problem (11.57±4.15). Similarly, those who had topical fluoride applications by dentist had significantly (p=0.003) lower mean caries scores (8.67±4.14) than those with no topical fluoride application (11.9±2.89).

**Conclusion:**

The studied CP children had very high caries experience and poor oral hygiene. There was strong association between the high caries experience and poor oral hygiene.

The term cerebral palsy (CP) encompasses a group of permanent disorders of the development of movement and posture, causing activity limitations that are attributed to non-progressive disturbances occurred in the developing fetal or infant brain.^1^ Population-based reports show that prevalence of CP worldwide ranges from 1.5 to more than 4 per 1,000 live births.^2^ A prevalence rate of 23.4/10,000 live births has been reported in the Kingdom of Saudi Arabia.^3^ The CP children are highly vulnerable to various dental diseases including dental caries.^4^,^5^ The high vulnerability to dental caries in these children has been attributed to numerous factors including challenges in maintaining optimal oral hygiene, use of soft and carbohydrate-rich diet and lack of appropriate dental care.^6^,^7^ Previous and recent studies in CP children have reported high dental caries prevalence among these children.^5^,^8^ In view of the high rate of CP in the world and the high vulnerability of these children to dental caries, it is important to regularly gauge dental caries level and related risk factors in these children. Information is scarce in health-related scientific literature regarding oral health of CP children. The purpose of the present study was to gather latest information on dental caries experience, oral hygiene status and dietary caries risk factors in Saudi CP children.

## Methods

In this cross-sectional study, children were randomly selected from all the CP children registered in the 2 main centers for special children in Riyadh, Kingdom of Saudi Arabia; the study was conducted from December 2014 to May 2015. Sample size was determined considering the analytical plan, level of confidence (α=0.05) and margin of error 0.5 resulting in a sample size of 52. Every third child in the list of all the registered CP children in the 2 centers was selected for the clinical examination. Inclusion criteria were; cerebral palsy children, both genders, age 16 years or younger, and registered in the 2 main centers for special children in Riyadh. Exclusion criterion was; the children suffering from medical conditions other than those normally associated with CP. The medical conditions were confirmed from the children’s medical files in the 2 centers.

The World Health Organization criteria were used for the diagnosis of dental caries.^9^ Oral hygiene was assessed using the oral hygiene index described by Nanda.^10^ All the children were examined by one researcher, a senior pediatric dentist. Approximately 20% of the sample was re-examined for determining intra-examiner reliability, which was found to be 0.93. A self-administered questionnaire in Arabic was developed for the study. The questionnaire was modified from the one utilized in a previous study.^11^ The questionnaire was pre-tested for validity and reliability in 20 parents of CP children not participating in the main study. There was a time interval of 2 weeks for the test-re-test reliability. Appropriate changes were made to improve the questionnaire’s comprehensibility for the participating parents. The following information was collected from parents through the questionnaire: Demographic information such as parent’s age/gender; Child’s age, gender and CP type (from the child’s medical record); Importance of good dental health for optimal general health of the CP children; Oral hygiene practices in the CP children; Frequency and reason for dental visits of their CP children; Importance and various sources of fluoride for CP children and Dietary practices, frequency and type of foods/drinks used in main meals and snacks for the CP children.

The study was registered with King Saud University College of Dentistry Research Center (CDRC). Ethical approval was obtained for the study including the questionnaire utilized in the study. The study was conducted in the 2 main centers for special children in Riyadh City where health care and education is provided to the children with various conditions/disabilities such as cerebral palsy and Down’s syndrome. The CP children were examined in the centers’ dental clinics. These dental clinics are meant to provide palliative dental care and regular 6 monthly preventive visits to the CP children. The questionnaires were handed over to parents for completion. All the questionnaires had a covering letter and a consent form explaining the research objectives and ensuring anonymity/confidentiality of the information obtained through the dental examination and the questionnaire.

The collected data were entered into the computer utilizing Statistical Package for Social Sciences version 19 (IBMCorp, Armonk, NY, USA). Various frequencies were derived. Intra-examiner reliability was determined through Kappa statistics. A t-test was utilized to determine any significant difference (*p*≤0.05) in mean DMFT scores in relation to various independent variables. Normality of these variables was tested using Shapiro-Wilks Test. Multivariate Step-wise Regression analysis was conducted to identify a useful subset of the predictor independent variables for mean DMFT scores. Pearson Chi-Square and Fisher’s Exact Test were used to determine any correlation between oral hygiene status and the CP children’s gender and age group. Step-wise regression analysis was conducted to explore which predictors (independent variables) seem to provide a good fit.

## Results

A total of 52 CP children were examined. Mean age of the CP children was 6.3±2.7 years, ranging from 3-10 years (male 61.5%, female 38.5%). Mean parental age was 34.2±7.5 years ranging from 20-55 years (mothers 86.5%, fathers 13.5%). The majority (92.3%) of the children were spastic, and the rest had ataxia (7.7%). The CP children were divided into 2 age groups; 3-6 years (57.7%) and 7-10 years (42.3%) for further analyses.

### Dental caries

Only one (1.9%) child out of the 52 examined had no clinical caries. The combined (dmft and DMFT) mean caries score among the study sample was 9.98±3.99. There was no significant (*p*=0.382) difference in the combined mean caries scores between the male (9.59±3.84) and female (10.6±4.24) CP children. However, the older children had significantly higher (*p*=0.017) mean caries (11.5±3.34) score then younger children (8.86±4.1). Data were also analyzed in terms of mean caries scores in primary teeth (dmft) and permanent teeth (DMFT). The mean dmft score of the sample was 8.28±4.02 with a mean decay component of 6.9±3.73, missing component of 0.4±1.1 and filled component of 0.98±2.0. The mean DMFT score was 1.69±2.72 with a mean decay component of 1.59±2.55, missing component of 0.07±0.55 and filled component of 0.01±0.13.

Data were further analyzed for mean caries scores in primary teeth in younger (3-6 years) children and permanent teeth in the older (7-10 years) children. The mean dmft score was 8.56±4.27 in younger children, while the mean DMFT score in older age group was 3.59±2.78.

### Oral hygiene

Majority of the children had either fair (55.8%) or poor (34.6%) oral hygiene. Only one in 10 (9.6%) children had good oral hygiene. There was no significant difference in oral hygiene status in terms of gender (*p*=0.995) or age group (*p*=0.281) of the children (**Table 1**). Similarly, no difference was found in CP children’s oral hygiene status in relation to their parent’s gender *p*=0.855 or age (*p*=0.676).

**Table 1:** Oral hygiene status of CP children according to gender and the age group.

CP Children	Oral Hygiene Status			Total
	Good	Fair	Poor	
	n (%)			
***Gender***
Male	3 (9.4)	18 (56.2)	11 (34.4)	32 (100)
Female	2 (10.0)	11 (55.0)	7 (35.0)	20 (100)
*Total	5 (9.6)	29 (55.8)	18 (34.6)	52 (100)
***Age group***
3-6 Years	4 (13.3)	18 (60.0)	8 (26.7)	30 (100)
7-10 Years	1 (4.5)	11 (50.0)	10 (45.5)	22 (100)
^†^Total	5 (9.6)	29 (55.8)	18 (34.6)	52 (100)

* p-value=0.995,

† p-value=0.281, CP - cerebral palsy

### Dental caries & oral hygiene

Oral hygiene and dental caries correlated clearly, as the CP children with good oral hygiene had the lowest mean caries score 5.8±7.32, followed by those with fair 9.72±3.3 and poor oral hygiene 11.55±3.05. These differences in mean caries score were statistically significant (*p*=0.012).

### Other preventive factors and dental caries

Out of the large number of independent factors such as dental visits, use of fluoride, and dietary practices that were analyzed with respect to a possible association with dental caries, only the ones with a relevant effect are being listed in **Table 2**. The children whose first visit to dentist was for a routine check-up had lower mean caries score as compared to those who made first dental visit due to some dental problem. The CP children with topical fluoride application by a dentist also exhibited lower mean caries scores than those without. Potato chips as snacks were a marker for higher caries levels, as were bottled juices during main meals. Step-wise Regression analysis model confirmed privation of topical fluoride application by a dentist and use of bottled juices as significant predictors for high caries scores (*p*=0.001).

**Table 2:** List of independent variables affecting the caries scores in the CP children

Independent variables	Caries Score Mean±SD	*P*-Value
***Reason for first dental visit of the CP Child?***		
Routine Check-up	7.33±4.65	.028
Dental Problem	11.57±4.15
***Topical Fluoride application by a dentist?***		
Yes	8.67±4.14	.003
No	11.9±2.89
***Used potato chips as snack?***		
Yes	10.93±3.09	.053
No	8.78±4.69
***Used bottled juices during snacks?***		
Yes	10.71±3.89	.061
No	8.8±3.95

CP - cerebral palsy, SD - standard deviation

## Discussion

The study has fielded useful information regarding caries experience and risk factors in CP children on whom literature is scarce. However, the study had some challenges and limitations. Dental caries is a multi-factorial disease and it is not possible to encompass all these factors and sub-factors in one study. This study therefore focused on specific factors. In addition, dental examinations are a challenging task in CP children due to pronounced neuro-muscular incoordination in these children. Thus, such studies in CP children require personnel trained and experienced in doctoring these children. The parents of these children are often hard to reach and already very occupied with different health problems faced by the CP children, making it difficult to get consent for participation in studies. The present study overcame these challenges through use of a compact well-designed questionnaire, and conduction of a focused dental examination by an experienced person within an accustomed environment. The parents who participated in this study knew that oral health care workers were conducting the study. Therefore, possibility of favorable responses by the parents to various oral health-related items in the questionnaire could not be ruled out, and be considered as a limitation of the study.

### Dental caries

Almost all the studied children had clinical dental caries with very high caries levels which are well above the already high Saudi Arabian caries values.^12^ Although studies in various parts of the world mostly have reported high caries experience among CP children,^5^,^13^,^14^ the caries experience in this study with a mean caries score of 9.98 dmft/DMFT is the highest. In the present study, caries values increased with age indicating a persisting caries activity, mostly due to the cariogenic oral environment being characterized by mostly suboptimal oral hygiene. Most of the caries were still untreated due to the challenging setting for restorative treatment in CP children. This is also partially due to the patients and their caretakers seeking professional treatment too little and too late.^15^ Special expertise or structural equipment such as GA facilities are required for adequate dental treatment in these medically compromised children. Such a high treatment need requires enhancement of restorative dental services for these children through training of skillful workforce and creating more special dental care facilities.

### Oral hygiene

Very few children in the present study had optimal oral hygiene, which is in agreement with previous reports on oral hygiene in CP children.^8^,^16^ Chewing, swallowing and complete clearance of food from mouth are all challenging tasks for CP children. The CP children mostly are unable to perform routine oral hygiene procedures due to neuro-muscular incoordination and are usually dependent on their caretakers for performing these tasks.^7^ The lack of knowledge among the caretakers about how to perform oral hygiene procedures and on the importance of maintaining good oral hygiene, usually results in poor oral hygiene in these children. Even if the parents are aware of the importance of maintaining good oral hygiene in these children, most of them are so overwhelmed by the various challenge of taking care of their CP children that their dental health becomes a low priority.^13^,^17^ Nevertheless, sufficient daily oral hygiene is a prerequisite for optimal oral health.

### Dental caries & oral hygiene

This study confirmed the strong correlation between dental caries and oral hygiene. The CP children with poor oral hygiene had the highest mean caries score, confirming that groups without regular tooth brushing such as ECC children, social risk groups or institutionalized elderly exhibit high levels of oral morbidity.^18^ This clear association continues to be reported both in healthy and non-healthy individuals.^19^,^20^

### Other factors affecting dental caries

Preventive dental visits for a routine check-up and topical fluoride application clearly result in comparatively lower caries, which indicates potential ways for improvement in CP children’s oral health. Other studies have reported similar results.^5^ A six-monthly or even more frequent dental check-up visit in these children also offers an opportunity to the dentist for providing preventive information and instructions to the caretakers of these children. This can be the first step to ensure sufficient daily oral hygiene.

Fluorides produces significant caries reductions.^21^ Besides the topical fluoride applied in the dental office,^22^ the daily home use of fluoridated toothpaste is essential for good oral health in these children as this accounts for most of the caries decline in many populations.^23^ However, extreme care has to be taken in application of top fluoride in these children, considering their neuromuscular incoordination.

Historically; bottled juices usually have been shown to be acidic in nature and have added sugar, thus creating high potential for dental caries.^24^ Recent studies have pointed out towards high cariogenic potential of these juices (Mahajan et al 2014).^25^ Bottled juices were one of the 2 predictors for high caries scores in this study, as shown by the regression analysis model. It has long been recommended to limit the use of these acidic and sweetened juices in children.^26^ These juices could be especially harmful for CP children’s teeth that already have compromised oral environment due to the reasons already narrated above.

Crispy potato chips as between-meals snacks are a clear marker for highly cariogenic diet.^27^ In CP children, such food could be especially harmful due to its sticky nature, remaining in the mouth for a longer periods. In addition, its consumption is usually accompanied by sweetened and acidic drinks, which are usually highly cariogenic in nature. An insufficient oral hygiene status and a highly cariogenic diet result in very high caries levels in CP children which calls for appropriate actions to allow equal chances for good oral health in these children, who are already in a challenging situation due to their primary medical background. Poor oral health and, low quality of life due to oral health problems in these children could be prevented with similar strategies that have made the caries decline possible in other populations.^28^,^29^ An early dental check-up visit assists dentists and parents in timely identification of weak areas, and to provide education as well as instructions for the caretakers in home dental care of the CP children. The regular routine dental check-up visits ensure reinforcement of the preventive efforts, allow additional fluoride applications and support early and easier interventions in case of the disease development.

In conclusion, the studied group of CP children exhibited very high caries levels (mean dmft/DMDT score of 9.98±3.99. Only one in ten (9.6%) CP children had good oral hygiene.There was a strong correlation between poor oral hygiene and high dental caries experience (*p*=0.012).
